# Peptide-Based Bioconjugates and Therapeutics for Targeted Anticancer Therapy

**DOI:** 10.3390/pharmaceutics14071378

**Published:** 2022-06-29

**Authors:** Seong-Bin Yang, Nipa Banik, Bomin Han, Dong-Nyeong Lee, Jooho Park

**Affiliations:** 1 Department of Applied Life Science, Graduate School, BK21 Program, Konkuk University, Chungju 27478, Korea; tjdqls414@gmail.com (S.-B.Y.); baniknipa@gmail.com (N.B.); bm15@kku.ac.kr (B.H.); des7735@kku.ac.kr (D.-N.L.); 2 Center for Metabolic Diseases, Konkuk University, Chungju 27478, Korea

**Keywords:** peptide, drug conjugate, prodrug, drug design, anticancer therapy, bioconjugate

## Abstract

With rapidly growing knowledge in bioinformatics related to peptides and proteins, amino acid-based drug-design strategies have recently gained importance in pharmaceutics. In the past, peptide-based biomedicines were not widely used due to the associated severe physiological problems, such as low selectivity and rapid degradation in biological systems. However, various interesting peptide-based therapeutics combined with drug-delivery systems have recently emerged. Many of these candidates have been developed for anticancer therapy that requires precisely targeted effects and low toxicity. These research trends have become more diverse and complex owing to nanomedicine and antibody–drug conjugates (ADC), showing excellent therapeutic efficacy. Various newly developed peptide–drug conjugates (PDC), peptide-based nanoparticles, and prodrugs could represent a promising therapeutic strategy for patients. In this review, we provide valuable insights into rational drug design and development for future pharmaceutics.

## 1. Introduction

Peptide-based drug design and delivery could offer several advantages, such as high selectivity, low immunogenicity, and a convenient manufacturing process [1]. Since insulin, the first therapeutic peptide, appeared on the market in 1923, it has achieved remarkable results in the management of various diseases [2]. In the global clinical market, more than 100 peptide-structure-based drugs are used as pharmaceutics in therapy [3,4]. A functional peptide itself can, in general, be used as a drug without any modification; however, most peptides are administered to patients in forms maximizing the therapeutic effects via a combination of drug-delivery systems or molecular modifications [4,5]. For example, peptides such as insulin have various molecular structures with different drug formulations. Thus, various insulin drugs are sold worldwide, with a value of over USD 30 billion [6]. Considering their role and advantages in physiological activity, peptide-based drugs are expected to be widely used in the future [7].

In anticancer treatment, peptides are being developed for various therapeutic or diagnostic purposes [8,9,10]. Approximately 10 million patients are currently suffering from cancer, and they are usually treated with chemotherapy, radiation, and surgical resection to eliminate tumors [11,12]. In the case of chemotherapy, strong chemotherapeutics such as doxorubicin (DOX), paclitaxel, gemcitabine, and cisplatin are used to eliminate tumors, exhibiting cytotoxicity toward cancer cells. Combined with peptides, there are many ways to reduce the side effects of cytotoxic drugs on non-malignant cells, improving the effectiveness of cytotoxic agents [13,14]. Targeting a selective cell-membrane receptor or tumor-site biomarker with a peptide ligand or peptide-based molecules can allow the payloaded or conjugated drug to reach the tumor site at the highest concentration [15,16].

There are many different approaches to using peptides for anticancer therapy. Some small peptide-based inhibitors can show therapeutic effects by inhibiting cancer-specific enzymes or proteins overexpressed in tumor cells. For example, programmed death-1 (PD1) and programmed death-ligand 1 (PD-L1)-inhibitory peptides have been developed to block the activity of PD-L1, which is overexpressed in tumor cells [17]. These peptides show strong efficacy comparable to that of commercial cytotoxic anticancer drugs in animal models [18]. In the clinical market, there are peptide-based anticancer drugs that are currently used due to their strong efficacy. For example, leuprolide is a peptide drug that targets the gonadotropin-releasing hormone receptor, and it is used in the treatment of prostate cancer, while goserelin is a clinically available synthetic peptide drug for treating breast and prostate cancers [7]. These peptide drugs were approved in the 1990s and are still widely used.

An interesting use of peptides as therapeutics in cancer treatment is peptide–drug conjugates (PDCs). When PDCs are well designed using target-specific peptides and strong cytotoxic drugs, a small molecular cancer-specific prodrug or tumor-specific nanoparticles can be utilized for therapy. Interestingly, the delivery of cytotoxic drugs bound to peptides in PDCs is often compared to antibody–drug conjugate (ADC) delivery systems. Both antibodies and peptides can serve as the targeting ligands, so cytotoxic drug conjugates are able to show tumor-specific therapeutic effects or site-specific delivery via covalent bonds [19,20]. Although large numbers of ADC biomolecules linked to antibodies have been extensively developed in the clinical field, therapeutics based on peptides have advantages over ADCs. For example, several peptide-based bioconjugates are able to form nanoparticles (NPs) through self-assembly, etc., and these nano-sized molecules can show enhanced permeability and retention (EPR) effects at the tumor site [21,22]. In addition, they have the potential to overcome the limitations of ADC in the future because they can deliver significantly more drugs to tumors than antibodies. Therefore, peptide–drug conjugates have recently emerged as anticancer drug candidates throughout pharmacology [23,24,25,26,27].

Biomolecules based on peptides have various potential functions to utilize anticancer agents for therapy. From that point of view, we will highlight the current limitations of peptides for clinical application and their improvement as advanced cancer therapeutics in this review. At first, the fundamental problems and critical biological barriers of peptides need to be addressed for the successful development of new peptide drugs. These biological barriers are in many ways similar but different from protein-based or small synthetic anticancer drug development. Then, we summarized therapeutic or functional peptides and peptide derivatives that can induce apoptosis, immune response, and self-assembly, thereby increasing the anticancer effect. Additionally, various peptide and drug conjugates can function as targeting molecules towards tumors, and self-assembly for nanomedicines or prodrugs can be selectively cleaved by tumor enzymes. This will be discussed to consider how peptide-based drugs can be applied to cancer treatment.

## 2. Barriers Associated with the Administration of Peptide Therapeutics

Peptides studied as anticancer candidates have faced several obstacles in their further development into clinically available drugs. Most drugs are usually administered orally or parenterally; however, in the case of peptides, parenteral administration is preferred due to peptide stability problems [28]. The route of administration of peptide therapeutics is mostly limited to parenteral injection, as it is very difficult to administer them orally due to their rapid disintegration and low stability. In addition, some peptides can be administered through the skin or subcutaneously, but their clinical application in the clinic is very rare. In this review, we will not discuss all administration routes of peptides except (intravenous) parenteral injections, because the most clinically available peptide-based medicines are formulated for injection only [29]. Parenteral injection of peptides-based biomolecules represents the most common route of administration; however, it also faces various biological barriers in the blood circulatory system. From the perspective of the development of peptide-based therapeutics, physiological barriers need to be overcome to develop peptide-based biomolecules for anticancer therapy.

Intravenous administration of peptides for anticancer therapy usually face biological barriers in the blood, which must be overcome [30]. In the presence of serum or plasma, proteases/peptidases easily destroy or degrade peptides, resulting in proteolytic instability in the body. When a peptide drug is parenterally administered to a cancer patient, it does not have a long half-life due to its low stability in vivo; this is one of the reasons why there are few peptide drugs for anticancer therapy. Moreover, most peptide drugs are not highly permeable, so that can be a biophysical barrier to the target site (tumor). In anticancer therapy, few functional peptides have anticancer activity for a long time to kill cancer cells in the human body. Within 2~30 min, most peptides are easily cleared from the blood by the liver and kidneys [31]. To overcome the stability problem of peptides in vivo, some researchers tried to use a specific type of peptide that is resistant to decomposition. For example, D-peptides composed of D-amino acids can be anticancer peptides resistant to enzymatic degradation with long half-lives. Some proteolysis-resistant D-peptides of the immune checkpoint PD-L1 showed remarkable therapeutic potential for cancer immunotherapy. These peptides not only disrupted the PD-1/PD-L1 interaction for immune response in the tumor microenvironment but also were highly resistant to proteolysis in human serum [18]. These kinds of peptide derivatives may provide novel peptide-based drug candidates, overcoming physiological obstacles in the body.

**Figure 1:**
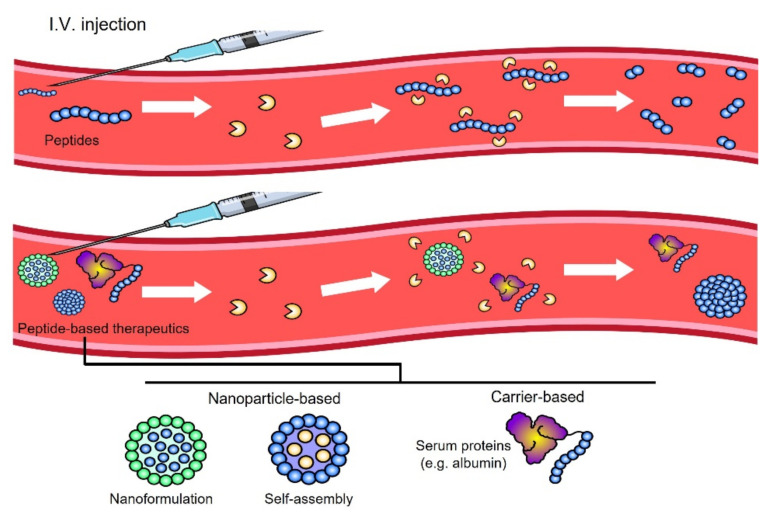
Proteolytic degradation of peptide products in the blood is considered a critical problem for clinical use. Peptide-based therapeutics such as peptide-based nanoparticles or carrier-utilizing peptide conjugates may stabilize peptide molecules in the circulatory system in vivo.

Researchers have tried to improve the blood stability of peptide-based therapeutics by utilizing many technologies, and a representative example is nanoformulations. The encapsulation of peptide-based products in nanoparticles can improve their blood stability, which is an inherent problem (Figure 1). Recently, self-assembled peptides or nanoparticles have been attracting attention in drug development for anticancer therapy, showing improved blood stability and tumor targeting effects. This type of nanotechnology has been attracting attention because it can overcome the limitations of peptide-based therapeutics while utilizing the biological function of peptides. In addition, various strategies have been introduced, such as a method to increase blood stability and the targeting effect using other proteins, including albumin or globulin. In particular, an albumin-based peptide delivery system can enhance peptide stability within the circulation, leading to enhanced antitumor efficacy [32]. The main purpose of using nanotechnology or carriers with peptides is to overcome blood stability issues, which are a fundamental problem for peptide-based molecules, meaning that many nano- or carrier-based drug candidates are currently being evaluated for conversion from laboratory investigations to clinical applications.

## 3. Peptide and Antibody Therapeutics

Low affinity toward their targets and metabolic instability due to enzymatic degradation are some of the drawbacks of peptides compared to antibodies [31]. Compared to developing an ADC, it is necessary to develop a given peptide with a clear understanding of its advantages and disadvantages in order to succeed in peptide-based drug development. Although many ADC-based drug candidates are being developed, there are some key properties of peptide–drug conjugates that warrant attention. The molecular size of an antibody in an ADC is larger than that of peptide-based biomolecules. For example, immunoglobulin G (IgG) antibodies contain more than 1000 amino acids (~150 kDa), whereas cancer-targeting peptides range from 5 to 25 [33]. Due to the large molecular size of an ADC, ADC-based therapeutics commonly face difficulties in their cellular uptake, half-lives in the plasma, immunogenicity, manufacturing costs, and stability [34]. On the contrary, small-sized (2~5 kDa) peptide-based drug conjugates can be synthesized by homogeneous entities and large-scale production. Additionally, several highly permeable peptides can penetrate the blood–brain barrier (BBB) in several cancer types [35,36,37]. There are many peptide-based biomolecules that are small and contain tissue-penetrating peptides. They generally exhibit superiority in tissue penetration and drug delivery.

**Figure 2:**
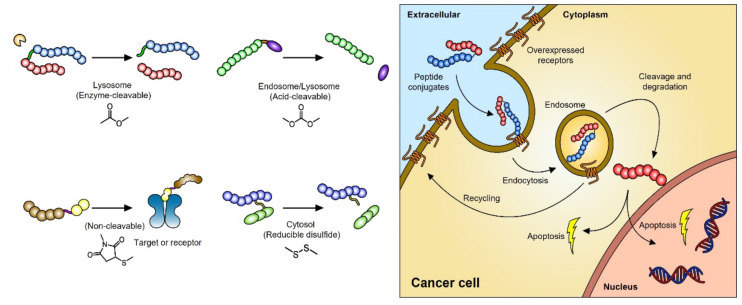
Due to the excellent function of peptides, they are often combined with a cytotoxic drug via a chemical linker in anticancer therapy. A number of functional and selective linkers can be used differently to design new therapeutics. These bioconjugates are usually taken up into cells by endocytosis and then cleaved by enzymes or other substances to release the cytotoxic agents.

In their molecular structures, peptide- and antibody-based drugs usually have a specific molecular linker in common. An ADC, a humanized or human monoclonal antibody conjugated to a small drug through chemical linkers, is a common therapeutic format in cancer chemotherapy [38]. Using a specific linker can improve the efficacy of a conjugated drug and can reduce its systemic toxicity. Therefore, a specific molecular linker needs to possess sufficient stability in the blood so that conjugated drugs can circulate in the body, showing high potency toward cancer with low off-target toxicity. These linkers include those that can be cleaved by lysosomal enzymes, those that can be cleaved by endosomal acidity, those that cannot be chemically cleaved, and disulfide bonds (Figure 2).

## 4. Peptides for Anticancer Therapy

### 4.1. Apoptosis-Related Peptides

**Figure 3:**
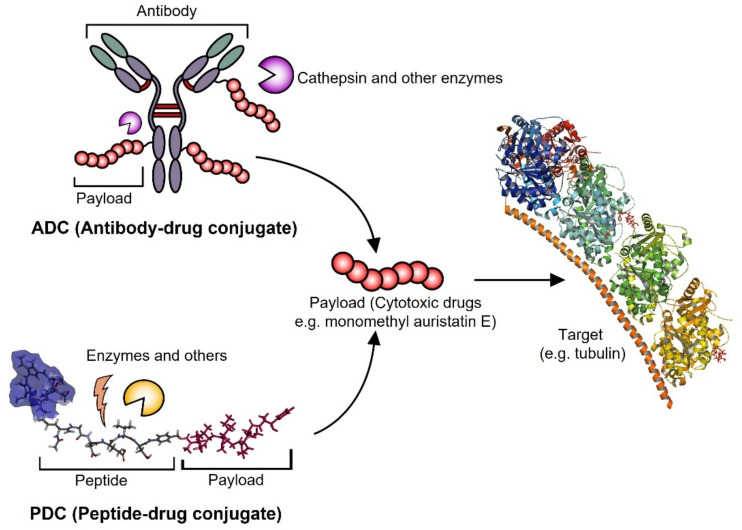
The concepts of antibody–drug conjugates (ADCs) and peptide–drug conjugates (PDCs) are fundamentally similar in drug-delivery systems. Strong cytotoxic drugs such as monomethyl auristatin E (MMAE), doxorubicin, and paclitaxel can bind to antibodies or peptides for targeting effects. In this case, conjugated MMAEs are released at the tumor site in anticancer therapy and then inhibit the same target (tubulin, PDB: 5IYZ) [42], leading to apoptosis in cancer cells.

Several peptides can induce apoptosis in cancer cells. For example, an anticancer peptide named Ra-V (deoxybouvardin) induces mitochondrial apoptosis followed by the loss of the potency of the mitochondrial membrane. The latter releases cytochrome C due to the destruction of mitochondrial the membranes in tumor cells. As a result, cytochrome C-induced apoptosis follows the activation of caspase in breast cancer cells [39]. Another pentapeptide, Dolastatin 10, is capable of inducing apoptosis by upregulating cytochrome C and Bax, which act as a proapoptotic molecule downregulating the expression of Bcl-2 [40]. In 2011, the FDA-approved, Dolastatin 10 derive, monomethyl auristatin E (MMAE)-fabricated drug Adcetris^®^ was constructed by linking it with a peptide linker (Valine citrulline) to an antibody to treat anaplastic large T-cell systemic malignant lymphoma and Hodgkin lymphoma [41]. Another example of an apoptotic peptide-related biomedicines is a DEVD (Asp-Glu-Val-Asp) peptide based bioconjugate. Recently, a DEVD peptide and MMAE conjugate, photosensitizer chlorine e6 (Ce6)-DEVD-MMAE, was synthesized and evaluated in in vitro and in vivo experiments. In the study, caspase-3 could be released by the molecular action by visible light with a photodynamic therapy (PDT) agent (Ce6) in cancer cells. The released apoptotic caspase-3 cleaved the DEVD peptide and induced sequential apoptosis, showing a strong therapeutic effect with minimized systemic toxicity [14]. As described above, the same cytotoxic molecule, MMAE, can be designed as an ADC or PDC, with advantages and disadvantages (Figure 3). In general, PDCs, which are nanoparticles based on self-assembly, can deliver large amounts of MMAE. On the contrary, ADCs can be more selective toward cancer cells due to the specific molecular binding of antibodies.

KLA (KLAKLAKKLAKLAK) is an interesting proapoptotic polypeptide that induces programmed cell death by disrupting the mitochondrial membrane. Bioconjugates incorporating KLA peptides can be applied to the development of various anticancer therapeutics. For example, KLA can be conjugated with iRGD (CRGDKGPDC), a tumor-homing peptide, to improve the penetration of low-grade tumor tissue and cells. The high tumor selectivity and low systemic toxicity of the recombinant KLA–iRGD conjugate have been confirmed in mice [43]. This type of research may lead to the development of new targeted PDC. Another example of KLA-based therapeutics is a penetratin (Pen; a cell penetrating peptide)-KLA conjugate. To overcome the low cell permeability of KLA peptides, penetratin was conjugated to KLA peptide via a disulfide bond, resulting in high cell permeability, and cytotoxicity even at low concentrations, as well as a strong apoptotic effect on mitochondria tubular organization that resulted in aggregation, unlike unconjugated KLA [44]. The conjugate has little effect on the mitochondria of normal cells, so it shows therapeutic potential as a new peptide-based drug conjugate.

### 4.2. Conjugates for Tumor Accumulation

Various methods for targeting tumors with peptides have recently been introduced. For example, Redko et al. demonstrated that short non-RGD (arginine-glycine-aspartate) S-S-bridged cyclic peptide (ALOS-4) had specificity for integrin avβ3, which is overexpressed in human metastatic melanoma. ALOS-4 is a peptide newly synthesized by Yacobovich et al. that does not contain canonical RGD sequences (H-cycle (Cys-Ser-Ser-Ala-Gly-Ser-Leu-Phe-Cys)-OH) [45]. In this study, ALOS-4 and a topoisomerase I inhibitor, camptothecin, were conjugated. As a result, the cytotoxicity of the conjugate was increased in human metastatic melanoma cells and decreased in normal cells [46]. In addition, Brunetti et al. conjugated the tetra-branched peptide NT4 with paclitaxel [47] based on their previously studied peptide [48]. This study showed that NT4 peptide can selectively bind to tumor-membrane-sulfated glycosaminoglycans. When it is conjugated with paclitaxel, it has strong selectivity against cancer cells, and this conjugation enables it to demonstrate more effective tumor regression compared to paclitaxel alone [47,49]. EphA2 is also an interesting protein related to cancer metastasis, which is overexpressed in various cancer cells such as melanomas, ovarian, prostate, lung, and breast cancers. Salem et al. conjugated dimeric 123B9 peptide, targeting EphA2, with paclitaxel. This peptide is an optimized variant of YSA [YSAYPDSVPMMS], an EphA2-targeting peptide, from a previous study of theirs. It can inhibit lung metastasis in breast cancer models [50].

Albumin is an interesting target in terms of tumor targeting and drug delivery in chemotherapy [51,52]. It is an endogenous protein that can circulate in the blood for longer than other plasma proteins. Therefore, it does not have toxicity or immunogenicity in the body, and it is taken up by rapidly growing tumor cells as a source of amino acids and energy [53]. Yousefpour et al. conjugated a protein-G-derived albumin-binding domain (ABD) with doxorubicin via a pH-sensitive linker [51]. This study showed that the ABD–DOX conjugate has a longer half-life in the plasma than DOX alone, and it showed four times higher accumulation in tumors. In another study, the tumor-targeting effect of an albumin-binding peptide was investigated. In the in vitro study, albumin-binding-peptide (DICLPRWGCLW)-based bioconjugates formed stable albumin complexes for tumor-targeting effect [52]. They also showed high tumor-targeting efficacy in a SCC7 tumor-bearing mouse model with an increased half-life [52]. In addition, in their latest paper, Kim et al. developed a novel peptide–drug conjugate that could utilize albumin metabolism [54]. Based on the positive correlation between albumin uptake and tumor growth, albumin-binding could be a promising therapeutic method for targeting tumor tissues.

### 4.3. Nanomedicine in Peptide Therapeutics

Drug delivery systems with nanomedicine in peptide therapeutics have become increasingly important in recent years. Peptide-based self-assembled or functional nanoparticles show high potential due to their excellent efficacy and low toxicity. For example, in anticancer therapy using peptides, curcumin is widely used in terms of targeting and nanoformulation. Curcumin, a polyphenol derived from Curcuma longa (turmeric) root, is known as a promising anticancer candidate. However, there are some limitations to further developing it as an anticancer drug on its own. It has low water solubility and bioavailability and is rapidly cleared from the bloodstream. Recently, Hasanpoor et al. overcame these obstacles by encapsulating curcumin with albumin [55]. Then, they conjugated a programmed death ligand 1 (PDL1)-targeting peptide, RK-10, to the nanoparticles. The RK-10 peptide was synthesized according to a sequence previously reported by Caldwell et al. [GSGSGSTYLCGAISLAPKAQIKESL] [56]. Their bioconjugate formed nanoparticles and showed significantly increased cytotoxicity toward PDL1-overexpressing breast cancer cells (MDA-MB-231). Inducing nanoparticle construction with self-assembling peptides can direct drugs toward tumors by EPR effect. Peptides, which are affected by different environmental conditions, assemble themselves with unique structures and act as biomedical materials. Self-assembling peptides thus have some different functional possibilities according to their divergence in structure. They can be used as scaffolds for the regeneration of cells and tissues or carriers for delivering drugs and can be employed to target the controlled release of stable drugs with less toxicity. Moreover, the reverse self-assembly of nano-carriers and nanomedicines in cells is a distinct property of these peptides [57]. In the process of peptide self-assembly, well-organized aggregates can be formed by selective peptides [58]. These self-assembled aggregate structures maintain a steady low-energy state due to hydrogen bonding, hydrophobic interactions, electrostatic interactions, and van der Waals forces [59]. In aqueous peptide self-assembly, intermolecular interactions are efficiently improved by aromatic moieties. This principle prioritizes π–π interactions that establish unique physical and chemical properties of self-assembled peptide nanomaterials [60]. Self-assembled nanostructures such as vesicles, nanotubes or nanorods, nanoparticles, and micelles form the core of research into drug delivery against cancer [61]. One such example is carrier-free small-sized peptide (Phe-Arg-Arg-Gly; FRRG)-conjugated doxorubicin-based nanoparticle. These amphiphilic peptide conjugates form a stable self-assembling nanoparticle structure under aqueous conditions without any synthetic nano-sized carriers [15]. This type of self-assembling method can solve the many problems of current nanoparticle-based drug-delivery systems. The use of self-assembled peptides takes advantage of their preferential accumulation in tumor tissues due to EPR effect. The development of self-assembly peptide-based nanomedicine can promise highly effective anticancer therapy options for cancer patients.

### 4.4. Cancer Immunotherapy

Attempts to treat cancer by controlling the function of immune cells are also in the spotlight. Programmed cell death ligand (PD-L1) is usually overexpressed on various cancer cells and, combined with programmed cell death protein 1 (PD-1; CD279), which is overexpressed on activated T cells, enables the immune evasion of cancer cells. The inhibition of PD-1/PD-L1 interactions is a promising target in cancer immunotherapy. Among the immune-related peptides, D-PPA [NYSKPTDRQYHF] is a PD-L1-binding peptide developed by Chang et al. Their study demonstrated that the hydrophilic D-type polypeptide (D-PPA) itself did not have cytotoxicity; however, through blocking the PD-1/PD-L1 interaction, it could inhibit tumor growth and prolong animal survival in CT26 tumor-bearing mice [18]. In other studies, Zhu et al. made a conjugate-based PD-L1-binding nanoparticle with doxorubicin [62]. A hydrophilic D-PPA polypeptide was linked with two hydrophobic stearyl chains via a pH-sensitive linker to form amphiphilic PD-L1-binding peptide conjugate (DCS)-based nanoparticles. The nanocarrier itself showed no toxicity and better internalization than doxorubicin alone. Furthermore, when studied in a colon cancer CT26-bearing mouse model, the significantly increased antitumor effect of the doxorubicin-loaded nanocarrier was confirmed with the detection of enhanced CD8+ cell activity in tumor tissue.

Melittin is an interesting peptide for cancer immunotherapy with further therapeutic applications. Melittin accounts for approximately 50% of bee venom, and it is a linear peptide composed of 26 amino acids. According to PubChem’s database, its sequence is [GIGAVLKVLTTGLPALISWIKRKRQQ]. Many studies have shown that melittin has anti-inflammatory, antiviral, and anticancer activities. As a bioconjugate for anticancer therapy, Liu et al. synthesized melittin with interleukin 2 (IL-2) [63]. IL-2 is cytokine mainly secreted from CD4+ T cells but it is also secreted from CD8+ cells and natural killer (NK) cells, activating dendritic cells. It is known as one of the most effective cytokines in immuno-cancer treatment; however, a high concentration of IL-2 can cause systemic toxic effects [64]. The study used mutant IL-2 (MIL-2) to improve the antitumor effect and reduce its systemic side effects. The melittin–MIL-2 bioconjugate functioned similarly to MIL-2 itself, but with a greater cytolytic effect. It increased the production of interferon (IFN)-γ in various cancer cells originating from liver, breast, ovary, and intestinal tissue. It also inhibited tumor growth and prolonged the survival time compared to the control and melittin alone in MIL-2 treated groups in a SMMC-7721, A549, SKOV3 tumor-bearing mouse model.

Macrophages are immune cells that play an important role in innate and adaptive immunity. Once activated, macrophages start to fight pathogens such as microorganisms, abnormal cells, and foreign substances [65]. Macrophage activation has two opposite states, M1 (classical) and M2 (alternative). In the tumor microenvironment, macrophages sometimes promote tumor immunosuppression, and these are called tumor-associated macrophages (TAMs). The colony-stimulating factor 1/colony-stimulating factor 1 receptor (CSF-1/CSF-1R) pathway is related to macrophage survival and differentiation. As CSF-1R is considered a promising target for cancer immunotherapy, a peptide named M2pep [Cys-YEQDPWGVKWWY] was modified in a recent study to increase specificity to target M2 TAMs. Li et al. confirmed that co-encapsulating with CSF-1R siRNA and M2pep can target M2 TAMs for reprograming to the M1 type, leading to increased antitumor immune responses [66]. As a result, the activated antitumor immune reaction showed a synergistically therapeutic effect in a pancreatic cancer animal model.

## 5. Bioconjugates with Drugs

With the concept of a drug–drug conjugate, a combination of peptides with cytotoxic drugs or other therapeutic drugs can be utilized for the development of peptide-based therapeutics. Commonly, cytotoxic drugs that are capable of inhibiting tumor cell growth or cell function are used in anticancer therapy. Unmodified cytotoxic drugs are prone to inhibiting normal fast-dividing cells in the body, rather than only cancer cells [48]. In various studies, these cytotoxic drugs can be combined with peptides with an additional function. For example, among the anticancer therapies, platinum-based cisplatin chemotherapy is one of the mostly widely used strategies for cancer treatment. However, the long-term clinical use of cisplatin in patient reduces its therapeutic effect because of drug resistance. The use of peptides may be important as a method with which to solve drug-resistance-related problems. For example, Li. et al. demonstrated that cisplatin- and peptide-combined nanoparticles could improve the cellular uptake of cisplatin and decrease its systemic toxicity. In the study, cisplatin-loaded modified dextran nanoparticles were designed specifically to bind to the luteinizing hormone-releasing hormone (LHRH) receptors overexpressed on the surface of 4T1 breast cancer cells [67]. The peptide conjugate induced high accumulation of cisplatin in tumor and metastasis-containing organs. It also significantly reduced the systemic toxicity of cisplatin.

Some other non-cytotoxic drugs in clinical use can be designed for peptide-based cancer therapy. Disulfiram (DSF) is an interesting FDA-approved anti-alcoholism drug that can inhibit aldehyde dehydrogenase (ALDH). Although it was not developed for chemotherapy, several studies have reported that DSF has anticancer effects, such as inducing apoptosis in cancer and decreasing the angiogenic effect of cancer cells [68]. Using several nanotechnologies with DSF, He et al. conjugated diethyldithiocarbamate (DDTA), a metabolite of DSF, with a polymer named poly[(2-(lycosyl-2-yldisulfanyl) ethyl acrylate)-co-[poly (ethylene glycol)]] (PDA-PEG) [69,70]. They also added lactobionic acid (LBA), which is a well-known β-d-galactose receptor ligand that can increase specific interaction with the targeted receptor. Compared to that in the control group, tumor growth was inhibited in a peritoneal metastatic ovarian tumor model when their LBA-PDA-PEG-DSF (LDNP) polymer was applied as treatment. They also confirmed that the anticancer effect was increased with additional copper ion treatment. Fluvastatin is statin used to treat hypercholesterolemia and to prevent cardiovascular disease, approved by the FDA in 1999. Several studies have reported that this drug may have anticancer effects, inhibiting protein glycosylation. From this point of view, Badr-Eldin et al. synthesized a bioconjugate with bee venom peptide (melittin) and fluvastatin (FLV-MEL) [71]. This FLV-MEL conjugate showed the strongest cytotoxic effect and changed the cycles of OVCAR3 ovarian cancer cells compared to those in the group treated with melittin or fluvastatin alone. Additionally, the FLV-MEL conjugate could increase proapoptotic activity, demonstrating its synergistic anticancer activity. Other recent studies have reported that cyclooxygenase-2 (COX-2) is overexpressed in cancer cells, along with a decrease in peroxisome proliferator-activated receptor γ (PPARγ) expression. Celecoxib is a COX-2-selective nonsteroidal anti-inflammatory agent (NSAID) approved by the FDA and widely used as a treatment for osteoarthritis and rheumatoid arthritis. In a recent study, Uram et al. conjugated celecoxib and FMOC-L-Leucine, a PPARγ agonist with biotinylated polyamidoamine (PAMAM) dendrimer of generation 3 (G3). The conjugated and biotinylated G3 PAMAM dendrimers conjugated with both drugs (celecoxib and FMOC-L-Leucine, 1:1) showed higher cytotoxicity than the drugs alone or single-drug conjugates [72].

There are peptide conjugates that are currently being studied clinically for the treatment of intractable tumors. On 27 February 2021, melflufen (melphalan flufenamide, PEPAXTO^®^), an allogeneic first-in-class peptide–drug conjugate, was approved by the US Food and Drug Administration (FDA) for the treatment of multiple myeloma, a disease where monoclonal plasma cells in the bone marrow grow uncontrollably and cause overproduction of immunoglobulins or immunoglobulin chains [73]. As melflufen is highly lipophilic, it can be easily taken up by targeted cancer cells. Once internalized, melflufen molecules can be cleaved by aminopeptidases, which are highly expressed in multiple myeloma cells, releasing their alkylating agents. This tumor-specific action afforded by the peptide conjugation can cause critical DNA damage and apoptosis in tumor cells [74]. As another example, Lutathera (lutetium Lu 177 dotatate) was approved by the FDA for the treatment of somatostatin receptor (SSTR) positive gastro-entero-pancreatic neuroendocrine tumors on 26 January 2018 [75]. Somatostatin receptors (SSRTs), especially subtype 2 (SSRT2), are highly expressed in gastro-entero-pancreatic neuroendocrine tumors (GEP-NET) in the stomach, small intestine, rectum, and colon [75,76,77]. Lutathera has a high affinity for somatostatin subtype 2 receptors (SSRT2). Lutathera is internalized by cancer cells, and the beta emission from Lu 177 leads to the formation of free radicals, which, in turn, can lead to cellular damage in SSRT2-overexpressing cells [75].

Prodrug strategies using peptides with a conjugated ester bond that can be broken down by esterase have recently been studied for anticancer therapy [78]. For example, ANG1005 (paclitaxel trevatide) is a newly developed peptide–drug conjugate consisting of three molecules of paclitaxel with an angiopep-2 peptide [79]. This paclitaxel-based bioconjugate was designed to treat brain tumors and is able to overcome the blood–brain barrier (BBB). As a result, ANG1005 can cross the blood–brain barrier by targeting low-density lipo-protein receptor-related protein 1 (LRP1), which is highly expressed in the BBB. LRP1 is a receptor protein related to tumorigenesis; however, its molecular binding with ligands (or peptides) can induce receptor-mediated endocytosis for cellular uptake. Upon binding to LRP1, ANG1005 can then be internalized by LRP1-mediated endocytosis. Then, lysosomal esterase can cleave the peptide backbone of ANG1005, releasing the cytotoxic paclitaxel, which kills the cancer cells. Esterase is a ubiquitous enzyme that hydrolyze small ester-containing molecules, yielding hydroxyl and carboxylate groups. Prodrugs development strategies that utilize such esterase-mediated cleavage of an ester group are steadily attracting attention.

In terms of targeted cytotoxic approaches using cytotoxic drugs, bicycle drug conjugates (BDCs) have therapeutic potential with strong antitumor activity. Among tumor microenvironment (TME)-associated proteins, membrane type 1-matrix metalloprotease (MT1-MMP) is a protein that is overexpressed in cancer cells, promoting cell migration [80]. Considering the up-regulation of MT1-MMP in cancer, it is a potential drug target for treating cancer. BT1718, a bicyclic peptide constrained to MT1, targets MT1-MMP in various cancer cells, including lung cancer [81]. Because MT1-MMP is mainly overexpressed in cancer cells, normal cells can be less affected by BT1718. BT1718 has been shown to have in vivo antitumor effects on a tumor-bearing mouse model, allowing the drug’s progression to phase two of a clinical trial [81]. BT8009 is another bicycle peptide–toxin conjugate targeting nectin-4 for treatment of malignant tumors [82]. BT8009 consists of a conjugated bicyclic peptide joined to the antimitotic toxin monomethyl auristatin E (MMAE) via a sarcosine spacer chain and cleavable linker. As is well known, the synthetic antineoplastic tubulin inhibitor MMAE has high toxicity; thus, it cannot be used alone. Because BNectin-4 is a protein involved in cell–cell adhesion and is present at high levels in various malignant tumor cells [83,84], BNectin-4-targeting BT8009 can bind to Nectin-4 on breast cancer MDA-MB-468 cells and kill them [85]. The therapeutic effect and toxicity of BT8009 have been evaluated in clinical trials. In addition, MMAE can be targeted to cells via metastasis-related proteins such as EphA2. EphA2 is a receptor that is overexpressed in various cancer cells. BT5528, a bicyclic peptide–MMAE conjugate, can target EphA2 and then release cytotoxic MMAE in tumor cells. Bennett et al. demonstrated that BT5528 had high cytotoxicity toward tumors and showed rapid renal elimination [86].

**Figure 4:**
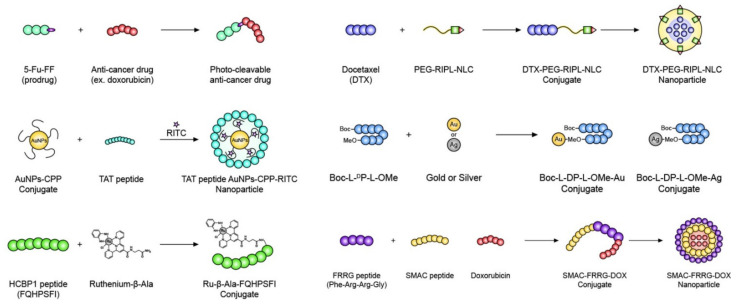
Several functional peptide-based drug carriers that have recently been actively studied.

Various other recently developed peptides or peptide-based conjugates are used with anticancer agents for the treatment of cancer, as summarized in Figure 4 and Table 1. Many peptide-based therapeutics or PDCs show improved biological functions and tumor-targeting effects for tumor therapy. These peptide therapeutics can outperform most conventional small anticancer molecules, antibodies, and ADCs. Some of the various biomolecules pursuing strategies such as drug conjugates, prodrugs, and nanoparticles should be moved forward onto clinical trials.

## 6. Conclusions and Perspectives

Due to the advantages of peptides, peptide drugs or peptide–drug conjugates have been widely explored for new drug development or drug delivery. Previously, peptide drugs were seen as merely mimicking hormones or endogenous peptides in the body, but they are now being reborn for the design of new therapeutics, giving them biological functions. Few clinical cases used peptides in anticancer therapy in the past. However, some specific functional peptides and PDCs are now in the spotlight as new biomolecules, for which various existing problems have been addressed. Conventional chemotherapy has several clinical problems to overcome in terms of efficacy and toxicity. Therefore, various therapeutic strategies applying cytotoxic anticancer drugs and functional peptides have been developed, outperforming small drugs and larger antibodies.

In this review, we summarize the recent successful progress in the preclinical and clinical development of peptide-based therapeutics, providing instructive insight. Because systemically administered peptides are naturally susceptible to degradation and physiological environments, delivery barriers and their related factors must be considered for successful peptide drug development (Section 2). It can be similar to the well-known protein-based drug development, but the physicochemical differences of peptide molecules must be taken into account (Section 3). Although various protein-based therapies have been developed in the clinical market, peptide-based therapeutics have clear advantages, such as their targeting effects and easy production. Some drawbacks of peptide-based therapeutics can be overcome by using functional peptides related to cancer immune therapy, apoptosis, and nanoformulation (Section 4), or with chemical modification of the selected drugs and peptides (Section 5) for enhanced therapeutic effect. Especially, when a designed peptide capable of tumor-selective cleavage or a tumor-targeting effect is successfully introduced into other drugs or small molecules, the low biostability and effect issues for peptides can be reduced. Among them, an interesting strategy is using self-assembled amphiphilic peptides for nanoformulation and immune modulating peptides for cancer immunotherapy. Peptide-based nanomedicines and immune modulators are becoming important with excellent therapeutic efficacy and tumor-specific effects, as well as low systemic toxicity in the field of anticancer therapy. These kinds of peptide-based therapeutics are expected to be able to overcome the difficulties of conventional anticancer therapy. Peptides or peptide-based bioconjugates will further draw widespread attention, providing rational guidance for research into the design of new drugs and smart drug-delivery systems.

## Figures and Tables

**Table 1:** Various peptide–drug conjugates for targeted anticancer therapy.

Peptide	Effects	Peptide-Conjugated Drug	Cell Line	Highlight	Year	Refs
FF dipeptide	Peptide-conjugated photo-cleavable linker	5-Fu-FF-COOH ^1^ (Prodrug)	Hela cell	Photo-controlled drug delivery	2019	[87]
AE147 ^2^	High anticancer effects for overexpressing uPAR ^3^	Docetaxel-AE147-PEGlyted Liposome NPs (Drug)	MDA-MB- 231, MCF-7	Low IC_50_ value (4.61 µg/mL) found for breast cancer cells	2021	[88]
Tet 213 AMP ^4^	Antimicrobial infection activity	ALG/HA/COL-AMP ^5^ Tet 213 (Dressing material)	NIH 3T3	A bioactive agent facilitating the proliferation of fibroblast cells	2019	[89]
Boc-L-^D^P-L-Ome	Anticancer activity against colon cancer	Boc-L-^D^P-L-Ome-Au(Ag)-NPs	MDA MB-231, HT-29	A superior effect on malignant tumors at a low concentration	2019	[90]
RIPL ^6^ peptide	Selectivity towards hepsin-expressing cancer cells	cPEG-RIPL-NLCs ^7^	SKOV3 and RAW 264.7	Sensitivity toward cellular uptake at pH 7.4	2018	[91]
^D^PPA	Prodrug improves immunotherapy	PEG/^D^PPA-MMP-DOX NPs ^8^	B16-F10	Co-delivery nanoplatform for improved chemoimmunotherapy	2021	[92]
RIPL peptide	Antitumor activity with high payload	DTX-PEG-RIPL-NLCs ^9^	SKOV3	Cell-cycle arrest observed in G2/M phase with apoptosis	2020	[93]
TP	Inhibition of cancer cell migration	DSPE-PEG-TP-NPs ^10^	MCF-10A MDA-MB-231	NKA a1-overexpressing cells inhibited by DSPE-PEG-TP-NPs	2021	[94]
Anti-Collagen IV	With a magnetic inner core	Fe_3_O_4_@Nanogels System-Col IV	A7r5 and HUVEC	Nanogel resulted in a controlled release of rapamycin	2018	[95]
TAT peptide	AuNPs-CPPs are distributed in bacterial strains	TAT peptide-AuNPs-CPP-FITC ^11^	Bacterial cells	Promising drug for multi-drug-resistant bacteria	2018	[96]
GE11 peptide	Anticancer activity with a synergistic combination	GE11-CUR/ICG-LPs ^12^	A549 cells	This peptide has a specific target receptor on EGFR	2018	[97]
HCBP1 ^13^	Act as a ruthenium based anticancer agent	Ru–β-Ala-FQHPSFI ^14^	Hep-G2 DDP)	Hepatoma-targeting peptide	2020	[98]
Amino peptides	Represent anticarcinogenic effects on breast cancer cells.	Melflufen	MDA-MB231	High efficiency observed due to lipophilic peptide-conjugated alkylator drug	2020	[99]
RIPL	PEG3000 at various ratios (1%, 5%, and 10%)	PEG(5%)-RIPL-NLCs	SKOV3 MCF7	PEG at a 5% molar ratio acts as a promising nanocarrier for hydrophobic drugs	2018	[100]
Pip8b2	Can recover from muscle-wasting disease	Pip8b2-conjugated splice-switching oligonucleotides	Myoblasts	Conjugation with the peptide improves exon skipping	2020	[101]
iWnt ^15^	Inhibits resistant breast cancer cells	iWnt-ATF24-IONP-Dox ^16^	MDA-MB-231	Nanoparticle drug with a property of dual-targeting Wnt/LRP and uPAR	2017	[102]
Deltorphan	Crosses the BBB and localizes in the CNS	PLGA: Glu-DP-PLGA: PLGA-CY5 ^17^	C57BL6J mice	The DP peptide, after entering the brain endothelial cells, stimulates endocytosis.	2020	[103]
TAT-peptide	Acts as an anti-SARS-CoV-2 therapeutic agent	TP ^18^-conjugated ritonavir, lopinavir, favipiravir and others	In vivo	They target SARS-CoV-2 main protease	2020	[104]
FRRG	NPs present proapoptotic activity	SMAC-FRRG-DOX-NPs ^19^	MCF-7 and others	Inhibit metastatic lung cancer with suppression of tumor growth	2020	[105]
R5K	Acts as an antiangiogenic agent	R5K-ITZ loaded PLGA-NPs	HUVECs ARPE-19	Physical stability at a specific temperature	2020	[106]
Cyclic RGD	A two-photon PDT agent	Ruthenium (II) complex-RGD	U87MG MCF-7	This tumor-targeting metallo-anticancer drug abrases mitochondrial integrin α_v_β_3_ rich cells	2020	[107]
RIPL peptide	Inhibition of tumor growth	DTX-loaded RIPL-NLCs	SKOV3 LNCaP	RIPL-NLCs demonstrate positively charged nanodispersion	2018	[108]
Stabilin-2 peptide (S2P)	Modified by S2P peptide containing imatinib	PLGA-Maleimide-PEG NPs containing Imatinib	VSMC	After 130 h, imatinib releases up to 100%	2020	[109]
TAT-peptide	Nucleus-targeting and imaging	DOX loaded TAT-IR780	4T1	Long-term fluorescence and photothermal imaging properties	2019	[110]
Tuftsin peptide	Inhibits the growth of HeLa cells	Dox-ALG-PEG-TFT, DOX/ALG-PEG-TFT	HeLa cells	Tetra-peptides induce phagocytosis and the immune system	2018	[111]
iRGD peptide	PLGA-SS-PTX/TET	iRGD-peptide mediate lipid polymer hybrid system	A2780	Cytotoxicity against MDR cancer cells	2017	[112]

5-Fu[29]-FF-COOH ^1^—5-fluorouracil-phenylalanine-phenylalanine-COOH; AE147 ^2^—KSD-cha-FskYLWSSK (cha—L-cyclohexyl alanine; s—D-form Ser; k—D-form Lys, acetate salt); uPAR ^3^—urokinase-type plasminogen activator receptor (uPAR); Tet 213 AMP ^4^—(amino acid sequence: KRWWKWWRRC) antimicrobial peptide; ALG/HA/COL-AMP ^5^—alginate/hyaluronic acid/collagen-AMP; RIPL ^6^—IPLVVPLRRRRRRRRC; cPEG-RIPL-NLCs ^7^—cleaved PEG-RIPL-nanostructured lipid carriers; PEG/^D^PPA-MMP-DOX NPs ^8^—PEG/antagonist of PD-L1- matrix metalloproteinases-doxorubicin NPs; DTX-PEG-RIPL-NLCs ^9^—docetaxel-PEG-RIPL-NLCs; DSPE-PEG-TP-NPs ^10^—1,2-distearoyl-sn-glycero-3-phosphoethanolamine-poly(ethylene glycol)_2000_-NKA αI-targeting peptide; TAT peptide-AuNPs-CPP-FITC ^11^—transactivating transcriptional activator-gold NPs-cell-penetrating peptides-fluorophore fluorescein isothiocyanate; GE11-CUR/ICG-LPs ^12^—GE11-curcumin/indocyanine green-liposomes; HCBP1 ^13^—FQHPSFI; Ru–β-Ala-FQHPSFI ^14^—ruthenium(II)-β-alanine-FQHPSFI; iWnt ^15^—NSNAIKNKKHHH; iWnt-ATF24-IONP-Dox ^16^—iWnt-amino terminal fragment 24-iron oxide NPs-doxorubicin; PLGA: Glu-DP-PLGA: PLGA-CY5 ^17^—poly-lactide-co-glycolic acid:lycosylated-deltorphin-PLGA:PLGA-cyanine 5; TP ^18^—TAT-peptide; SMAC-FRRG-DOX-NPs ^19^—SMAC-(Ala-Val-Pro-Ile-Ala-Gln*,* AVPIAQ)-FRRG-doxorubicin-NPs.

## Data Availability

Not applicable.
